# Towards Linking Histological Changes to Liver Viscoelasticity: A Hybrid Analytical-Computational Micromechanics Approach

**Published:** 2024-11-20

**Authors:** Haritya Shah, Murthy N. Guddati

**Affiliations:** North Carolina State University, Raleigh, NC 27695-7908

## Abstract

Motivated by elastography that utilizes tissue mechanical properties as biomarkers for liver disease, and the eventual objective of providing explicit links between histology and bulk mechanical properties, we develop a micromechanical modeling approach to capture the effects of fat and collagen deposition in the liver. Specifically, we utilize computational homogenization to convert the microstructural changes in hepatic lobule to the effective viscoelastic modulus of the liver tissue, i.e. predict the bulk material properties by analyzing the deformation of repeating unit cell. The lipid and collagen deposition is simulated with the help of ad hoc algorithms informed by histological observations. Collagen deposition is directly included in the computational model, while composite material theory is used to convert fat content to the microscopic mechanical properties. The results illustrate the model’s ability to capture the effect of both fat and collagen deposition on the viscoelastic moduli and represents a step towards linking histological changes in the liver to its bulk mechanical properties, which can provide insights for accurate diagnosis with elastography.

## 1. INTRODUCTION

Elastography, either based on MRI or US, measures the elastic modulus of tissue, and uses it as a biomarker for various diseases, including liver diseases such as liver fibrosis [1–3]. The fundamental premise is that disease causes microstructural changes, which in turn affects macroscopic tissue elasticity. Over the past decade, there is increased attention to viscosity of the tissue as a biomarker, in addition to elasticity, opening up diagnostic opportunities, e.g. for metabolic dysfunction-associated steatotic liver disease (MASLD) and metabolic dysfunction-associated steatohepatitis (MASH) [4–8]. The paper is aimed at linking the microstructural changes to mechanical properties, specifically in the context of the liver. While the proposed framework could be applicable to other diseases, we guide the development focusing on the MASLD, MASH and fibrosis. Further, the current investigation is motivated by wave-based elastography with small amplitude vibrations in the linear regime.

The histological changes through the evolution of liver disease are very complex. These include the effects of softening associated with fat infiltration as well as hardening associated with fibrosis, and the effects of nonlinearity associated with interstitial pressure and fat infiltration, poroelasticity associated with microchannel flow altered by inflammation and ballooning. While some of these effects can be captured through analytical models, such models will likely not be able to capture the interplay between the many complex phenomena. Motivated by this, this study presents the first step of developing a computational homogenization framework that captures the effects of lipid and collagen deposition. The resulting framework can then be gradually refined by adding various complexities after careful examination of histological and physiological processes, eventually resulting in a tool that can be used to quantitatively link histological changes to changes in mechanical properties. Thus the focus of the current paper is to develop such framework and perform preliminary illustration of the framework’s ability to capture the effects of steatosis and fibrosis.

While computational modeling to link microstructural properties to bulk mechanical properties of the liver has not been well explored, there are significant contributions in analytical and phenomenological approaches to provide these links. Parker and coworkers [9] used classical composite theory to quantify the effect of lipid deposition and validated with oil-in-gel phantoms. Conceptual link between histological parameters and mechanical models have been explored using animal models, which are not just limited to steatosis and fibrosis but also inflammation [4]. Finally, the correlation between fibrosis and liver stiffness has long been established where correlation between stiffness and fibrosis grades have been developed based on extensive research including *in vivo* studies [1, 3, 10, 11]. These studies are significant in independently addressing fibrosis and steatosis with respect to overall collagen and fat content respectively. They cannot be extended to include the effect of microstructural patterns of fat and collagen deposition, which may be a confounding factor in such correlations. Driven by this, and with the expctation that it can be gradually improved in complexity and precision, we propose a simple computational micromechanical model to capture the effects of steatosis and fibrosis.

Note that the proposed model is different from many existing microscopic computational models of the liver. For example, Wang and Jiang investigated shear wave behavior in liver microstructure by direct numerical simulation and not focused on the intermediate step of understanding homogenized (visco)elastic properties [12]. Ebrahem et al. investigated coupling poroelastic models with modeling blood flow in the liver and is not directly applicable to elastography investigations [13]. Yoshizawa et al. utilized agent based modeling to simulate the progression of fibrosis and not so much the effects of fibrosis [14]. We instead focus on relatively simpler question that is more relevant to elastography: what is the link between the fat and collagen contents and the bulk tissue viscoelastic properties?

To answer the question, we treat the liver to be an assembly of perfectly periodic hepatic lobules and apply computational homogenization to estimate the viscoelastic modulus for a given pattern of fat and/or collagen deposition. We also develop ad hoc microstructure generation algorithms to create synthetic microstructure profiles that mimic visual observations of histology. We then combine these two steps to present preliminary results for viscoelastic modulus as a function of fat content and/or collagen percentage area (CPA), duly noting that the presented results are from a framework that needs to be refined with data from real observations. Notwithstanding this, the framework is expected to serve as a stepping stone for such refinements.

The paper is organized as follows. After making some observations on liver structure and physiology, the Theory section summarizes the main approach as well as presents underlying governing equations and the computational homogenization approach that can link micromechanical properties to bulk moduli. The Methods section contains the approaches to generate the microstructure with fat and/or collagen, as well as the details of application of computational homogenization. The Results and Discussion section contains preliminary results for steatosis, fibrosis as well as combined steatosis and fibrosis, along with some observations based on the results. Finally the paper is concluded with some closing remarks.

## 2. THEORY

### 2.1 Liver Structure and Physiology

The mechanics and mechanobiolgy of the liver involve complex microstructure and physiological processes. Liver is composed of tree-like vasculature composed on large vessels (hepatic vein, hepatic arteries, portal vein and bile duct), branching multiple times to form smaller vessels (central vein and portal triad), which interact through the hepatic lobules. Given our focus on elastography, which is typically focused on regions away from large vessels, our current goal is to understand the deformation of locally homogeneous liver tissue made up of repeating hepatic lobules.

The hepatocytes, which form the large part of liver parenchyma interact mechanically with the microchannel flow within the sinusoids, making the process poroelastic [13]. The process is expected to be nonlinear due to the hyperplastic behavior of the parenchyma and non-Newtonian nature of the blood flow. Liver diseases change the microstructure due to e.g. lipid infiltration of hepatocytes, ballooning, inflammation, and collagen deposition, which in turn result in changes in the nonlinearity and poroelasticity. Fortunately, in the context of wave-based elastography, which is the motivation behind this work, the situation simplifies significantly. The high-frequency nature of wave deformation reduces the effect of flow-deformation interaction, effectively rendering the process (visco)elastic. Further, the small amplitude nature of the waves does not cause nonlinearity within the wave cycles, facilitating the probing of linearized viscoelastic properties of the current state of the liver tissue. Thus, the estimation of micromechanical changes in the liver from viscoelasticity measurements from elastography requires only linear elastic analysis of liver tissue away from the large vessels.

While the linearized material properties within a hepatic lobule may not be constant, as a first step, we will assume that the material properties are constant within the lobule (for the healthy liver without any lipid or collagen deposition). Furthermore, we do not explicitly model the effects of inflammation and ballooning and include them in the overall stiffness of the liver tissue; at this time, we only focus on the explicit effects of fat and collagen. Additionally, given that MRE typically uses 50–100 Hz frequency range and SWE focuses on the 100–300 Hz range, the wavelength is generally more than 3 mm. Given that typical lobule size is ~1 mm, while there may be some dispersion effects at the upper end of the frequency, these effects would not be significant, and intralobular wave scattering effects can be neglected as a first step. With such simplification, the viscoelastic properties of the bulk tissue in the liver can be computed from elastostatic analysis of repeating lobular structure, modified by lipid and collagen deposition. This is the focus of the remainder of the paper.

### 2.2 Overall Approach

Histological observations indicate that fat is deposited randomly within hepatocytes roughly as spherical inclusions. Parker et al. [9] utilized this observation and combined it with classical composite materials theory to provide analytical relationships between fat content and viscoelastic modulus. They approximate steatotic liver as a homogeneous tissue with random spherical inclusions of fat. Fat deposition, though random at the scale of hepatocytes, follow noticeable histological patterns at the scale of hepatic lobule. To account for this, we introduce a two-scale model of homogenization. Changes in localized viscoelastic modulus, at the intra-lobular scale, are predicted using the analytical formulation in [9]. This results in a varying distribution of viscoelastic properties within the hepatic lobule, which is then injected into a computational homogenization framework to predict the homogenized material properties of bulk liver tissue.

As an extension to studying the effects of combined steatosis and fibrosis that occurs sometimes e.g. in MASH, we introduce collagen deposition into the computational model. This conforms with the histological observations of MASH where repeated steatotic shocks to the liver tissue eventually results in some fibrosis. Given that the collagen length scale is larger than hepatocytes, we directly induce collagen deposition into the computational model of the hepatic lobule (as an initial step, this is done in an ad hoc manner to mimic histological observations). After examining the effect of collagen using this model, it is combined with the two-scale model for steatosis to provide some preliminary observations on combined steatosis and fibrosis, with some implications related to elastography.

In what follows, we will first summarize computational homogenization, followed by modeling steatosis, fibrosis, and their combined effect.

### 2.3 Computational Homogenization

**Figure 1:**
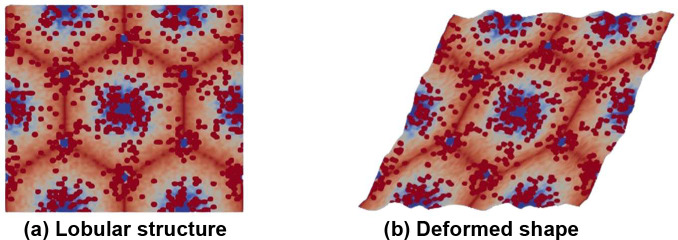
(a) Representative microstructure of the liver used in the current study (details of microstructure generation and analysis are presented later). The red spots depict average effects of collagen with high modulus, while blue regions are softer tissue due to fat deposition near central and portal veins. (b) deformed microstructure under imposed global shear strain, which not only contains the imposed global deformation but also the local oscillations in deformation due to microstructural heterogeneities

The basic idea of homogenization is to obtain material properties as experienced at macro scale, where the spatial scale of deformation is larger than the scale of the periodic cell, which is the hepatic lobule in the current study. Conceptually, homogenization is performed by applying macroscopic unit strain and examining the resulting tractions that can be converted to (averaged) macroscopic stress. Given the near-incompressibility of liver tissue at the microscale, the homogenized compressibility would also be near zero. We thus focus on obtaining the homogenized shear modulus. This process is schematically illustrated with Figure 1, while the underlying details of the microstructure generation and deformation analysis are explained in the remainder of the paper. The figure shows a square region of the original microstructure containing repeated liver lobules, and the deformed shape under applied homogenized unit shear strain. Only the global shear strain is specified, while locally the tissues deforms satisfying the equations governing viscoelastic deformation. The averaged homogenized stress is computed by analyzing the deformed shape with a framework known as asymptotic expansion homogenization (AEH, [15]). In what follows, we summarize the underlying equations as well as AEH within the context of viscoelastic deformation.

#### 2.3.1 Viscoelastic Deformation: Governing Equations

The deformation of the liver tissue is governed by equilibrium equation, stress-strain and strain-displacement relations. Equilibrium equation is given by,

(1) ∇⋅σ=f

where f is the body force vector, σ is the stress tensor. The stress σ is related to strain ε for a viscoelastic material through convolution integral:

(2) $$\sigma =\int_{-\infty }^{t}\;E(t-\tau )\left(\varepsilon (\tau )-\varepsilon_{0}\right)d\tau ,$$

where t,τ represent temporal variables, while E is the relaxation modulus tensor. $\varepsilon_{0}$ is the initial strain corresponding to the stress-free state. The strain is given by the symmetric gradient of the displacement u:

(3) $$\varepsilon =\nabla_{s}u.$$

General linear viscoelastic models for linear systems are most easily analyzed in the frequency domain, where the above stress-strain relation in Equation (2) takes a much simpler product form:

(4) $$\sigma (\omega )=E(\omega )\left(\varepsilon (\omega )-\varepsilon_{0}(\omega )\right)$$

where ω is the temporal frequency. Same notation is used for both time and frequency domains and the precise meaning is understood based on context. Assuming isotropy, the frequency-dependent complex modulus E(ω) can be written in terms of complex-valued, frequency-dependent bulk and shear moduli. Given the near incompressibility of soft tissues, the deformation is dominated by shear distortion, governed only by the shear modulus, i.e. $G(\omega )=G^{\prime }(\omega )+iG^{\prime \prime }(\omega )$, where $G^{\prime },G^{\prime \prime }$ are storage and loss moduli respectively. Near incompressibility is simulated by taking the Poisson’s ratio close to 0.5. With such an approach, the elastic modulus tensor can be written as $E(\omega )=E^{\prime }(\omega )+iE^{\prime \prime }(\omega )$, where $E^{\prime }$ captures the effect of the storage modulus and $E^{\prime \prime }$ captures the effect of the loss modulus.

Complex arithmetic is not directly implemented in most computational simulation software, including the one employed here, Multiphysics Object Oriented Simulation Environment (MOOSE, [16]). To facilitate the current simulation involving complex arithmetic, we convert the equations to real arithmetic separating real and imaginary parts of field and state variables, i.e. displacements, strains and stresses, i.e. $u=u_{r}+iu_{i},\varepsilon =\varepsilon_{r}+i\varepsilon_{i},\sigma =\sigma_{r}+i\sigma_{i}$. We similarly split the forcing function, $f=f_{r}+if_{i}$. Writing the variables in composite form, e.g. $\overset{-}{u}={\left\{u_{r},u_{i}\right\}}^{T}$, the equilibrium and strain-displacement relations in Equations 1,3 do not change in their form, except that the variables are replaced by the composite form of real and imaginary parts. On the other hand, the stress-strain relation, when expanded, takes the form:

(5) $$\left\{\begin{array}{l} \sigma_{r}\\ \sigma_{i} \end{array}\right\}=\left[\begin{array}{cc} E^{\prime } & E^{\prime \prime }\\ -E^{\prime \prime } & E^{\prime } \end{array}\right]\left(\left\{\begin{array}{l} \varepsilon_{r}\\ \varepsilon_{i} \end{array}\right\}-\left\{\begin{array}{l} \varepsilon_{0,r}\\ \varepsilon_{0,i} \end{array}\right\}\right).$$

The above viewpoint indicates that the viscoelasticity equations in the frequency domain can be solved using real arithmetic with expanded form of field variables, coupled with the expanded form of the modulus tensor represented by the bracketed term in Equation 4. We implement this expanded form through MOOSE inputs.

#### 2.3.2 Asymptotic Expansion Homogenization

Global (shear) strains on a periodic cell, needed for computational homogenization, can be applied with nonhomogeneous periodic boundary conditions. The resulting displacements can be processed for tractions on the boundary, which in turn can be averaged to obtain the global stresses. At the end this results in global stress-strain relations and thus the effective modulus tensor. A mathematically equivalent technique that is more convenient to implement and is readily available in MOOSE is the asymptotic expansion homogenization algorithm (AEH). AEH separates the total displacement to applied global displacement, and the local displacement which is the difference between the total displacement and the applied global displacement. The local displacement then satisfies simpler, homogeneous periodic boundary conditions. This perturbation is expanded in terms of Taylor series with respect to the size of the unit cell, and the low-order terms are equated to result in the effective modulus tensor. The final form of the effective homogenized modulus tensor $E^{H}$ takes the form:

(6) $$E_{ijkl}^{H}=\frac{1}{\left|Y\right|}\int_{\gamma }E_{ijkl}\left(I+\frac{\partial v_{k}^{mn}}{\partial y_{l}}\right)dy\;,$$

where Einstein’s indicial notation is used for convenience, with subscripts i,j,k,l representing the coordinate directions. Y is the unit cell, E is the modulus tensor that is variable within $Y.\;v^{mn}$ is the displacement variable from solving the governing equations with unit initial strain in mn direction, i.e. the superscripts m,n correspond to the two coordinate directions associated with the applied global strain. Given that only shear strain is applied in our setting, m=1 and n=2.

The process of AEH including solving for $v^{mn}$ and subsequent computation of $E^{H}$ using Equation 6, is available in MOOSE, once the governing equation associated with Equation 5 is implemented. This construct is utilized in the current work.

#### 2.3.3 Unit Cell

A thin slice of liver tissue, when viewed under a microscope, will appear to have a roughly repeating lattice made up of roughly regular hexagons with central vein at the center, and portal triads at each of the vertices. Though the primary repeating structure is hexagonal, given that AEH is derived and implemented in Cartesian system of coordinates, it is simpler to utilize a rectangular unit cell to apply the AEH procedure. To this end, we propose to use a unit cell shown in Figure 2, made up of four halves of adjacent hexagons. The diameter of the hepatic lobule is assumed to be 1 mm in diameter based on histological observations. This translates to the rectangular repeating unit having a width of 0.86 mm and a height of 1.5 mm by simple geometrical analyses. Note that the unit cell and all the analyses are two-dimensional. We do not consider three-dimensional volume because, (a) representing complex 3D packing of the lobules is complicated, (b) 3D simulations are computationally expensive, and (c) 3D packing of lobules is not completely well structured, making the precise treatment of the complex behavior from (a) and (b) would not be of much value, if any.

## 3. METHODS

### 3.1 Modeling Steatosis

#### 3.1.1 Intralobular fat distribution

To generate the distribution of viscoelastic modulus at the scale of multiple hepatocytes, we needed to first generate spatial distribution of fat, mimicking the progression of steatosis in an actual liver. Since there has not been extensive quantitative analysis of fat distribution as a function of disease progression, we start with an ad hoc fat distribution function with the idea of revisiting this should it become an important factor. With such an approach, the fat percentage is assumed to be linearly varying with the square root of the ratio of shortest distance from lobule boundary relative to the distance from the central vein:

(7) $$V=V_{min}+\left(\frac{\sqrt{x}\;-\;\sqrt{x_{min}}}{\sqrt{x_{max}}\;-\;\sqrt{x_{min}}}\right)\left(V_{max}-V_{min}\right),$$

where V is the fat content at the point in consideration, $V_{max}$ and $V_{min}$ denote the maximum and minimum fat fraction within the lobule, x represents the ratio of shortest distance from lobule boundary relative to the distance from the center of the central vein, and $x_{max}$ and $x_{min}$ are the maximum and minimum values of this distance for all points in the lobule.

**Figure 3:**
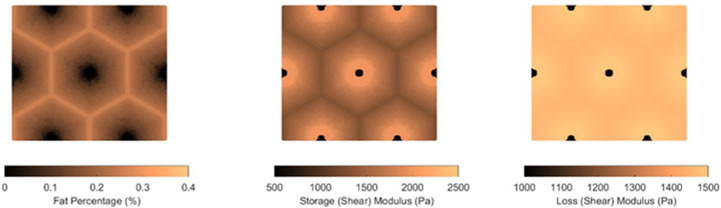
(a) Fat distribution for Pattern 2; (b) resulting storage modulus distribution; (c) loss modulus

Two patterns of fat distribution are considered. In Pattern 1, the fat content is the highest near the central vein, while in Pattern 2, the fat content is the highest near the boundary of the hepatic lobule. For both patterns, fat percentages are varied to control the total amount of fat present inside the hepatic lobule. The maximum fat percentage at any point within the lobule is capped at 50%, in line with the general histological observation of steatosis in the context of MASLD and MASH, and consistent with the mathematical limitation arising from Christensen’s theory of composite materials [17]. Lastly, given the random nature of fat deposition, 5% additive Gaussian noise was introduced to the fat distribution. Example fat distributions for both the patterns are shown in Figures 2a and 3a.

After populating the pixels in the domain of the unit cell with their respective fat concentration, the corresponding viscoelastic shear modulus is computed using analytical formulation using the formulas derived by Parker et al. [9]. This translates to a lobule with heterogeneous viscoelastic modulus distribution as shown in Figures 2b,2c,2b,3c. The goal now is to simulate the deformation of the unit cell under prescribed macroscopic strain and use it to obtain the homogenized modulus, which is done using AEH and finite element modeling.

#### 3.1.2 Finite Element Simulation

The unit cell in Figure 2 is discretized with 40×80 mesh of 8-node quadrilateral (QUAD8) finite elements, to capture the complicated displacement variation associated with heterogeneous structures. A 2×2 Gauss quadrature rule was used for numerical integration. The viscoelastic modulus at the integration points is obtained by the procedure described in the previous subsection. To verify that the accuracy of the results, mesh convergence analysis was performed. For a fixed fat content (20%), mean homogenized modulus from 100 different realizations was used to calculate error. The difference between the results from 40×80 and 80×160 meshes was found to be less than <0.5%. Hence, 40×80 elements mesh size was chosen for running the simulations.

### 3.2 Modeling Fibrosis

#### 3.2.1 Distribution of Collagen

Based on the inherently unstructured progression, fibrosis is simulated with a probabilistic approach informed by general observations from histopathology. The probability of collagen deposition at any given point is defined as:

(8) $$P=\Sigma \left(\left.\frac{x}{d}\right|\;\mu ,\sigma \right),$$

where x is the distance from a particular location (defined based on the type of fibrosis, discussed later), d is the control parameter that determines the spread of the deposition pattern and Σ is Gaussian integral, i.e. the cumulative distribution function associated with the normal distribution with mean μ (= 2) and standard deviation σ (= 10).

The definition of x depends on the type of fibrosis. For periportal fibrosis, x is taken as the distance to the closest portal triad, which has the effect of higher collagen deposition closer to the portal triad. For perisinusoidal fibrosis, x is taken as the distance from the closest central vein. For bridging fibrosis, the x is taken as the distance to the closest point to the edge where bridging occurs. If the bridging occurs between two portal triads, x is the distance to the hexagonal edge. For bridging between central vein and portal triad, the smallest distance from lobular radii connecting the central vein to the portal triads is used.

Actual fibrosis is not isolated to periportal or bridging patterns but exists as a combination of these patterns, determined by collagen proportionate area (CPA) as well as the deposition patterns. For stage 1 (F1, CPA ≤ 5%), we assume that only perisinusoidal fibrosis exists. For stage 2 (F2, 5% < CPA ≤ 10%), the collagen deposition beyond 5% CPA follows periportal pattern. Fibrosis deposition beyond 10% is assumed to follow purely bridging pattern.

The control parameter d is linearly interpolated based on CPA, from 0.75 mm at 1% CPA to 1.4 mm at 5% CPA, for perisinusoidal fibrosis occurring in stage 1. For stage 2 combining perisinusoidal and periportal fibrosis, d is set to 1.4 for perisinusoidal fibrosis and, for periportal fibrosis, it is linearly interpolated form 0.75 at 5% CPA and 1.75 at 10% CPA. For stage 3, d is linearly interpolated from 0.5 at 10% CPA to 1.0 at 20% CPA.

**Figure 4:**
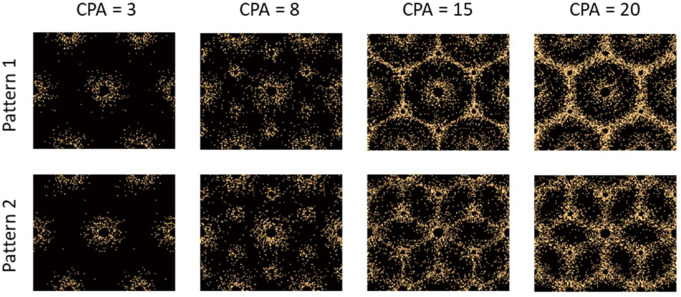
Realizations of two different patterns of collagen distribution inside a hepatic lobule with increasing CPA. Early-stage fibrosis in both patterns is the same. The differentiating in Patterns 1 and 2 based on bridging fibrosis starts with a CPA of 10% and higher. The yellow dots within the realizations are collagen fibers.

The deposition pattern as well as the parameters used in the deposition algorithm are necessarily ad hoc and are driven by visual comparisons of subsequently obtained realizations to observations of patterns from literature. It would be beneficial to make this more precise through quantitative analysis of existing histological observations, potentially complemented by simulations using mechanobiology; these studies are outside the scope of the current effort. Nevertheless, the proposed deposition algorithm results in realistic realizations of fibrosis at different CPAs are shown in Figure 4. As expected, the two patterns lead to the two different forms bridging fibrosis can take, starting at stage F3 and higher. Pattern 1 leads to bridging across the portal triads, while Patter 2 leads to bridging between the portal triads and central vein.

Given the realistic nature of the generated fibrosis patterns, we utilize them in the micromechanical model for AEH to examine the changes in viscoelasticity with respect to changing CPA. Unlike in steatosis where fat concentration needed to be converted into effective intralobular viscoelastic modulus, such conversion is not needed for fibrosis. Fat vesicles reside in hepatocytes but collagen deposition occurs at a larger scale around the hepatocytes and can thus be directly incorporated into the microstructural model.

#### 3.2.2 Finite Element Modeling

Unlike in steatosis where each point is assigned different viscoelastic moduli depending on the fat content, for fibrosis, each material point has a binary choice for viscoelastic modulus, either (viscoelastic) healthy liver tissue or (purely elastic) collagen. While such assignment appears simpler on the outset, it leads to sharp changes in material properties, leading to stress concentrations. Fortunately, since our goal is not to understand local stresses but to estimate the changes in macroscopic moduli, we can significantly simplify the problem. We start with the same mesh as steatosis, i.e. with 40×80 rectangular elements, which may be understandably coarse compared to collagen deposition pattern. We perform finite element analysis with this mesh, where the integration points are assigned material properties based on the procedure described in the previous subsection. The rationale behind this approach is that the implicit averaging performed through this approach would not lead to significant errors in macroscopic moduli which themselves are averaged quantities. Given that the homogenization is done over multiple realizations, we check this hypothesis by performing convergence analysis in a probabilistic sense. We essentially create 100 realizations of collagen deposition for 10% CPA and obtain the average viscoelastic modulus (4101+556i) kPa. We repeat the process but with a refined mesh of 80×160 elements, which resulted in an average modulus of (4064+555i) kPa. Given the error of ~ 0.89%, we concluded that 40×80 element mesh is sufficient for our current investigation.

### 3.3 Combined Modeling of Steatosis and Fibrosis

Steatosis and fibrosis can occur simultaneously in e.g. MASH, and it would be instrumental to examine the combined effects of CPA and fat content on both storage and loss moduli. This generalization of microstructure and associated finite element discretization is simple. Perform collagen deposition based on CPA, and for the remainder of the area, replace the healthy liver tissue by fatty tissue using algorithm in Section 3.1. Note that the average fat content needs to be adjusted by dividing with (1-CPA) as it is now the ratio of the volume of the fat to volume of the liver tissue not occupied by collagen. Once the intra lobular fat distribution is determined, the points occupied by collagen take the corresponding elastic modulus (taken as 60 kPa in this study), while viscoelastic modulus is assigned at the remaining points based on the approach in Section 3.1. At the end, like in the case of steatosis and fibrosis, we end up with a unit cell with varying modulus which is analyzed using AEH and FEM to compute global viscoelastic modulus. Just to confirm the accuracy of 40×80 finite element mesh, we obtained average viscoelastic modulus for 15% fat and 10% CPA, with 40×80 mesh, and with 80×160 mesh, which resulted in a difference of less than 2%. Thus the 40×80 mesh is deemed acceptable for simulating combined steatosis and fibrosis.

Statistically, about 90% of the patients develop pattern 1 of steatosis (fat concentrated in zone 3). Moreover, advanced-stage fibrosis (CPA > 10%) is accompanied by rapid loss of fat, hardening of the liver, and overall stiffening. Thus, guided by these histopathological observations, we choose to simulate only the combination of pattern 1 of steatosis, with fat fraction ranging from 0% to 30%, and early-stage fibrosis, with CPA percentages from 1% to 10%.

## 4. RESULTS AND DISCUSSION

### 4.1 Effect of Steatosis

**Figure 5:**
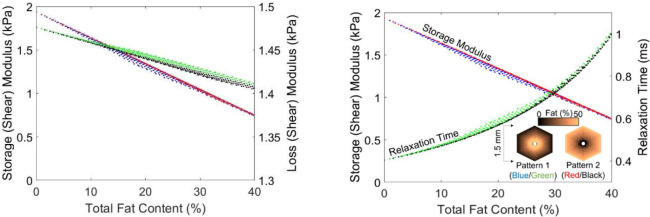
Plot of storage modulus and relaxation time vs. the total fat percentage for both the patterns of fat distribution.

The procedure described in Section 3.1 is utilized to generate different realizations of fat content distributions on the unit cell for varying overall fat content, for both Patterns 1 and 2. A total of 300 realizations are generated with fat content ranging from 0% to 40%. For these simulations, The viscosity of fat at body temperature is taken as 0.4 Pa.s, translating to complex modulus of G = 0.25i kPa at 100 Hz). Healthy liver tissue is assumed to have a shear modulus of 2 kPa and viscosity of 5 Pa.s, translating to complex modulus of (2+0.5i) kPa at 100 Hz. Figure 5a shows the variation of storage and loss moduli as a function of overall fat content (at 100 Hz). The deviation between the two patterns is most significant at the medium fat levels. In general, however, there does not appear to be much effect of the pattern of the fat deposition, compared to that of the overall fat content. An apparent clinical implication is that viscoelasticity can potentially be used to quantify the fat content in the context of simple steatosis, after appropriate investigation of in vivo effects.

As expected, the storage modulus decreases with increasing fat content, which is expected due fluidity of fat droplets. This is consistent with ex vivo observations that indicating that simple steatosis leads to softening of the liver tissue [18]. On the other hand, in vivo observations indicate that simple steatosis leads to increased stiffness of the liver [19, 20], which is attributed to the effects of in vivo confinement leading to increased pressure which in turn leads to increase stiffness. This can be captured with the help of hyper-elastic models, but such generalization is outside the scope of the current effort.

Figure 5a indicates that not only the storage modulus, but the loss modulus decreases with increasing fat content. While this may seem counterintuitive on the outset, it is reasonable in that the loss modulus of the fatty tissue is less than the loss modulus of the healthy tissue (=0.5 kPa). A more intuitive observation can be seen in Figure 5b where the results are plotted in terms of storage modulus and relaxation time τ, the ratio of effective viscosity to elastic modulus, i.e. $=G^{\prime \prime }/\omega G^{\prime }.\;\tau$ is essentially a measure of viscosity (relative to elasticity), and follows the expected trend: fat deposition increases the relative viscosity.

### 4.2 Effect of Fibrosis

**Figure 6:**
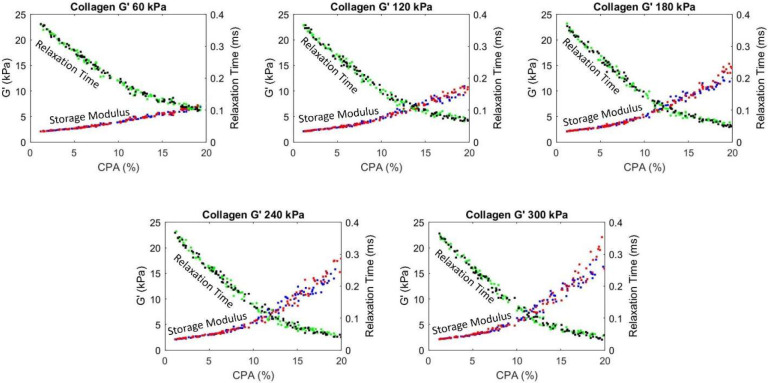
Plot showing the change in homogenized storage (shear, G’) modulus and relaxation time with increasing CPA percentage for various shear moduli (G’) of collagen. Blue and green are data points for Pattern 1 and red and black are for Pattern 2.

The procedure described in Section 3.2 is used to generate different realizations of collagen deposition inside the hepatic lobule, mimicking the progression of fibrosis across various stages. A total of 1,000 realizations with CPA ranging from 1% to 20% are generated. Similar to steatosis study in the previous section, the shear modulus of healthy tissue is taken as 2 kPa, and the viscosity is taken as 5 Pa.s. Given the modulus of the collagen is not well known, we consider various analyses with collagen shear modulus range identified from the literature, from 60 to 300 kPa, with an increment of 60 kPa. The resulting composite storage modulus and relaxation times are plotted as a function of CPA in Figure 6.

It is evident from Figure 6 that with an increasing CPA, the storage (shear) modulus increases and the relaxation time decreases. Moreover, for early fibrosis, up to 10% CPA, the scatter in the storage modulus as a function of CPA is not significant, indicating that there may be predictable relation between CPA and mechanical properties in the context of MASLD and MASH. Before using such relation with confidence, the collagen deposition patterns and modulus values should be determined more precisely and the results should be calibrated potentially with animal models to account for the *in vivo* effects not captured in the micromechanical model.

The plots in Figure 6 also show an accelerated increase in stiffening after CPA > 10%, especially for pattern 2 and for higher collagen modulus. This is attributed to the fact that the collagen deposition algorithm introduced structure to the pattern for CPA > 10%; as observed in Figure 4, 15% CPA has a clear structure facilitating load paths, indicating that collagen contributes significant increase in the effective modulus. This is immediately evident in Pattern 2 where the truss lattice structure of collagen rich areas facilitate stress flowing through these load paths. Pattern 1 has hexagonal structure, which still facilitates stress flow, albeit less efficiently than Pattern 2. In both cases, the stiffening is not just due to CPA, but also the structure of the collagen deposition pattern. Given the increased effects of the collagen, and given the random nature of its deposition, we also observe increased scatter of the predicted storage moduli. Note that significant quantitative conclusions should not be drawn from this study until careful investigation is performed into the collagen deposition patterns in real livers, as well as the representative modulus of collagen.

### 4.3 Combined Effects of Steatosis and Fibrosis: Preliminary Observations

As a preliminary study to examine the effects of combined steatosis and fibrosis, we repeat the simulations for 100 Hz, for moderate CPA (ranging from 1 to 10) and fat content ranging from 0 to 30%. Furthermore, we modify the healthy tissue properties to be power law {Parker, 2022 #386} with a power of 0.15 and a coefficient of 800 (units consistent with Pa). The collagen shear modulus is taken as 60 kPa and the fat viscosity is the same as before, 0.4 Pa.s.

**Figure 7:**
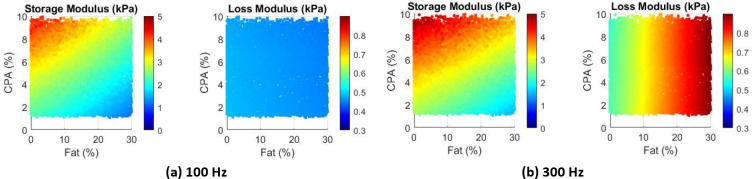
Distribution of storage modulus (kPa) and loss modulus (kPa) as a function of fat fraction and CPA fraction.

The contour plots for storage and loss moduli are presented in Figure 7a. As expected the storage modulus reduces with increasing fat, and increases with increasing CPA. Interestingly, the loss modulus does not change much; this is not unexpected with respect to CPA, as the collagen is not viscous and affects only the effective storage modulus and not loss modulus. The loss modulus does not change (much) even with changing fat content. Triggered by this, we repeated the simulation at higher frequency, 300 Hz, which can be considered as the upper end of ultrasound shear wave elastography. The results are presented in the right panel (b) of Figure 7. Clearly, both storage and loss moduli change with both CPA and fat content.

While the results in Figure 7 are preliminary, they can have important diagnostic implications. Looking at the specific case of Figure 7a at 100 Hz, given that only one quantity (storage modulus) is sensitive to fat content or CPA, it would not be possible to independently infer both CPA and fat content. On the other hand, using measurements at 300 Hz (Figure 7b), one can infer both CPA and fat content. While these models need to be revisited with careful inclusion of validated rheological models of liver tissue as well as elastic modulus of collagen in the liver, it appears that there is an important role frequency can play in estimating CPA and fat content from viscoelasticity measurements; ideally, it would be good to measure at multiple frequencies as done in ultrasound SWE and can be done in some versions of MRE.

## 4. CONCLUSIONS

As a first step towards building a model that can link changes in liver microstructure to changes in its mechanical properties, we develop a computational modeling framework to capture the effects of fat and collagen deposition. The model is based on a computational homogenization approach that simulates the viscoelastic deformation of repeating rectangular unit cell of the liver to predict the macroscopic storage and loss modulus at different frequencies. The collagen is explicitly included, while a smaller-scale analytical model based on composite material theory is utilized for modeling fat deposition. Ad hoc deposition patters are utilized to visually mimic collagen and fat content distribution within the hepatic lobule. With such patters, it is found that relatively coarse finite element mesh can obtain the effective modulus, implying the practicality of the approach even when multiple simulations are to be performed to study microstructural effects. We illustrated the framework to examine the effects of steatosis, fibrosis, and combined steatosis and fibrosis. Analysis of these preliminary results indicate that the mechanical changes due to steatosis are dependent on the total fat content, while the stiffening due to collagen is not only dependent on the overall content (CPA), but also the deposition pattern. Another interesting observation is that the frequency can play a role in relative sensitivity of CPA and fat content to the viscoelastic moduli. Before placing confidence in these studies, however, one must consider further enhancements described in Section 2.1.

## Figures and Tables

**Figure 2:**
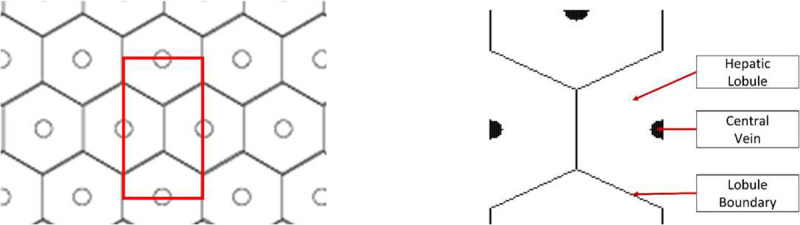
A 2D hexagonal lattice with the rectangular repeating cell highlighted in red and (b) layout of hepatic lobules in the identified rectangular unit cell.

**Figure 2:**
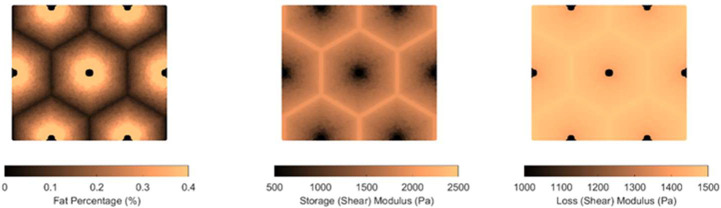
(a) Fat distribution for Pattern 1; (b) resulting storage modulus distribution; (c) loss modulus
